# Associations between obesity and sociodemographic, behavioral, and health characteristics in a population of women: a population-based study in Vitória, Espirito Santo, Brazil

**DOI:** 10.3389/fpubh.2025.1588877

**Published:** 2025-06-11

**Authors:** Nathália Miguel Teixeira Santana, Franciéle Marabotti Costa Leite

**Affiliations:** Lavisa, Department of Public Health, Federal University of Espírito Santo, Vitória, Brazil

**Keywords:** obesity, body mass index, sociodemographic factors, non-communicable diseases, depression

## Abstract

**Introduction:**

Obesity is a significant public health concern, with its prevalence rising in many countries worldwide. Studies have shown that women are more likely to become obese than men. This article aimed to describe the nutritional status of women, determine the prevalence of obesity, and explore its associations with sociodemographic, behavioral, and health characteristics.

**Methods:**

This cross-sectional, analytical, population-based study was conducted in Vitória, Espírito Santo, Brazil. It included women 18 years and older. Obesity was assessed based on self-reported weight and height and was classified according to the body mass index (BMI). Women were classified as obese (BMI ≥ 30 kg/m^2^) and non-obese (BMI ≥ 18.5 and <30 kg/m^2^). All analyses were conducted using Stata® 14.0 software.

**Results:**

Among the women studied, 3% were underweight [*N* = 32; 95% confidence interval (CI) 0.02–0.04], 41.8% were normal weight (*N* = 449; 95% CI 0.39–0.45), 33.2% were overweight (*N* = 356; 95% CI 0.30–0.36), and 22% were obese (*N* = 236; 95% CI 0.20–0.25). The prevalence of obesity was higher among women aged 30–39 years (27.6%), those with 0–8 years of education (33.9%), those in the lowest tertile of family income (30.5%), those who engaged in physical activity (26.7%), women with five or more pregnancies (39.7%), and those with diabetes (35.2%), hypertension (34.8%), and depression (29.7%) (*p* < 0.05). We found a significant association between obesity and all older age groups compared to the youngest one (18-29), except for women aged 60 or older (*p* = 0.004), who had a higher prevalence of obesity. Lower categories of education and family income registered almost twice the prevalence of obesity (prevalence ratio [PR]:1.70; 95%CI 1.21–2.38; *p* < 0.009 and PR: 2.00; 95%CI 1.36–2.95; *p* < 0.002) compared to those in the highest categories. Additionally, having five or more pregnancies was associated with an increased probability of obesity (PR: 1.66; 95%CI 1.04–2.64; *p* = 0.002). The presence of diabetes (PR: 1.34; 95%CI 1.03–1.74), hypertension (PR: 1.56; 95%CI 1.21–2.00), and depression (PR: 1.44; 95%CI 1.13–1.83) was also significantly associated with obesity compared to women without these diseases (*p* < 0.05).

**Conclusion:**

Our findings indicate that obesity is significantly associated with age, family income, education level, number of pregnancies, and chronic diseases, including diabetes, hypertension, and depression.

## Introduction

Obesity has become a significant public health issue due to its increasing prevalence in many countries worldwide (1). According to the World Health Organization (WHO) (2), individuals with a body mass index (BMI) equal to or greater than 30 kg/m^2^ are classified as obese. Despite its limitations in assessing body composition and fat distribution (3), the BMI has been widely utilized as a standard measure of obesity in epidemiological studies.

In Latin America, research has shown that women are more likely to become obese than men (4). In Brazil, the adult population has experienced a gradual increase in weight over the years. Between 2006 and 2012, a positive linear trend in weight gain was observed, with the increase being more prominent among women (5). Analysis of self-reported anthropometric data from Brazilian state capitals between 2006 and 2016 revealed an increase in the mean BMI of women, from 24.5 kg/m^2^ to 25.8 kg/m^2^ (6). A comparison between data from the 2002–2003 Household Budget Survey (HBS) and the 2019 National Health Survey (PNS) indicates that the prevalence of obesity among women nearly doubled, reaching approximately 30% in 2019 (7). In Vitória, Espírito Santo, a study conducted in 2013 estimated that 30.4% of the adult female population was obese, according to PNS data, while Vigitel reported a slightly lower figure of 16.1% (8).

Obesity, like many chronic non-communicable diseases, has a long latency period and is influenced by a range of factors, including genetic, socioeconomic, behavioral, and environmental determinants (9). Physical activity provides benefits both in the management of obesity and its associated diseases, as well as in the improvement of life quality (10). However, most associations with obesity are connected to harmful effects. It is commonly correlated with a high prevalence of hypertension, an increased risk of diabetes mellitus, coronary heart disease, myocardial infarction, neoplasms (11, 12), and depression (13). In addition, obesity contributes to a loss of years of life quality and an elevated risk of mortality from all causes (14).

Despite the multifactorial nature of obesity, it is a preventable condition. Understanding the factors contributing to its development is crucial for implementing effective prevention strategies. Therefore, this study aimed to describe the nutritional status of women living in Vitória, Espírito Santo, determine the prevalence of obesity, and explore its associations with sociodemographic, behavioral, and health-related characteristics.

## Materials and methods

### Type of study

This cross-sectional, analytical, population-based study was conducted in Vitória, Espírito Santo (ES), Brazil. The municipality of Vitória, the capital of Espírito Santo, had an estimated population of 322.869 people in 2022, a territory of 97.123 km^2^, and a municipal Human Development Index of 0.845. According to data from the 2022 census, the total female population of the territory represented 53.71% of the total population (173.415 women) (15).

### Study population and data collection

The research is part of a more extensive study whose main objective was to estimate the prevalence of intimate partner violence in adult women across the life course and during the pandemic. The study population comprised women selected through multi-stage cluster sampling based on census tracts and subsequent random sampling. Exclusive and shared households with at least one female resident were eligible for the study.

The sample size was calculated based on the census tracts of Vitória, using the number of existing household units according to the 2010 census, proportional to the existing number, with a larger number of interviews selected where there was a greater number of households. A random draw was conducted to conduct the interview in households with more than one female resident. Information of interest was collected during face-to-face interviews with the reference woman in each household. All interviews were conducted in a private place at their residence, without the presence of a partner. They were conducted only by female interviewers. The pilot study occurred in December 2021. The data collected in the pilot study were not part of the study’s final sample. Fieldwork began after data analysis of the pilot study.

Women 18 years of age and older interviewed were eligible. Women were excluded if they were incapable of understanding or communicating due to intellectual or sensory deficits and were, therefore, unable to respond to the instrument. The fieldwork took place from January to May 2022 with 20 interviewers and four field supervisors, totaling 1.086 women.

### Dependent variable

For this study, three women were excluded due to incomplete data regarding weight and/or height, and ten women were excluded due to BMI values greater than 3 standard deviations, generating a sample of 1.073 women. The presence of obesity was the study’s dependent variable and was measured based on self-reported anthropometric data of weight and height. BMI-classified nutritional status was calculated based on the formula BMI = weight (kg)/height (m2) expressed in kg/m2 and was based on the WHO classification proposal (2) as underweight, normal weight, overweight, and obesity. Subsequently, the women were classified as obese (BMI ≥ 30 kg/m2) and non-obese (BMI ≥ 18.5 and <30 kg/m2), with a final sample of 1.041.

### Independent variables

For sociodemographic variables, we used age groups in complete years (up to 29; 30–39; 40–49; 50–59; and 60 years or older), ethnicity/skin color (white and non-white), marital status (single or in a relationship for women who have their partners at the time of the interview, regardless of formal union), years of schooling (0–8, 9–11, and 12 or more), and household income in tertiles (the first tertile being the poorest and the third being the richest).

The behavioral variables were the ingestion of alcoholic beverages at least once in their lifetime (never, once a month or less, 2 to 4 times a month, 2 to 3 times a week, 4 times, or more a week), the practice of physical activity (yes or no), and current smoking (yes or no).

For the clinical variable, the number of pregnancies (none, 1 to 2, 3 to 4, and 5 or more), the presence of diabetes (yes or no), the presence of high blood pressure (yes or no), and the presence of depression (yes or no) were assessed.

### Ethical aspects

The Ethics Committee in Research with Human Beings approved the study at the Federal University of Espírito Santo under number 4.974.080 of September 2021. After clarification regarding the procedures to be performed, the participants signed the informed consent form (ICF), guaranteeing anonymity and the possibility of refusal to participate in the study.

### Statistical analyses

For this study, the prevalence of obesity in the sample was evaluated, and other association analyses were performed. All analyses used Stata® version 14.0 (Stata Corp., College Station, United States). A descriptive analysis was performed, presenting crude and relative frequencies. Bivariate analyses were performed using the chi-square test and Student’s t-test. Multicollinearity was assessed using the variance inflation factor (VIF), with a threshold of VIF < 5 considered acceptable. The possible mediation of obesity with the independent variables was explored using structural equation modeling (SEM). Poisson regression with robust variance was used to estimate crude and adjusted prevalence ratios (PR) and their respective 95% confidence intervals (95% CI). The variables associated with the outcomes under study were entered at levels according to the bivariate analysis (*p* < 0.20). The adjusted analysis was conducted by entering the variables into the model at three levels to account for possible confounding factors. In the first level, sociodemographic data (age group, race/color, education, family income, and marital status) were included; in the second, the data from the first model were supplemented with behavioral data (age group, years of schooling, household income, and physical activity). In the third model, the data from the first model were supplemented with clinical data (age group, years of schooling, household income, pregnancies, diabetes, hypertension, and depression). The final model adopted a significance level of 5% (*p* < 0.05).

## Results

In the 1.073 women studied, the mean BMI was 26.4 + −5 kg/m^2^, of which 3% were underweight (*N* = 32; 95%CI 0.02–0.04), 41.8% were eutrophic (*N* = 449; 95%CI 0.39–0.45), 33.2% were overweight (*N* = 356; 95%CI 0.30–0.36), and 22% were obese (*N* = 236; 95%CI 0.20–0.25). Among the obese, 166 (70.3%) were classified with class I obesity, 58 (24.6%) with class II, and 12 (5.1%) with class III. The prevalence of diabetes mellitus was 13.8% (95% CI 11.9–16.0), and hypertension was 27.3% (95% CI 24.7–30.1) in the sample.

The analysis presents the characteristics of women categorized based on the presence of obesity. Significant differences were observed between obese (*n* = 236) and non-obese (*n* = 805) women across several variables. Obese women had a significantly higher average weight (87.7 ± 10.7 kg) than non-obese women (64.3 ± 8.7 kg, *p* < 0.001). Obese women reported fewer years of education (10.9 ± 4.2) than non-obese women (12.5 ± 4.1, *p* < 0.001). Similarly, their average family income was significantly lower (3,298.41 ± 3,257 vs. 6,018 ± 7,825, *p* < 0.001). Physical activity was less prevalent among obese than among non-obese women (*p* = 0.001). Obese women exhibited significantly higher prevalence rates of diabetes (21.6% vs. 11.7%, *p* < 0.001), hypertension (42.8% vs. 23.5%, *p* < 0.001), and depression (24.6% vs. 17%, *p* = 0.009). However, no significant differences were observed for age, height, skin color, marital status, smoking, alcohol consumption, and number of pregnancies (Table 1).

**Table 1:** Characteristics of women according to the presence of obesity.

Variables	Non-obese (*n* = 805)	Obese (*n* = 236)	*p*-value*
	Mean ± SD	Mean ± SD	
Age (years)	46 ± 16	47 ± 15	0.675
Weight (kg)	64.3 ± 8.7	87.7 ± 10.7	<0.001
Height (cm)	1.62 ± 0.7	1.61 ± 0.7	0.265
Non-white (race/ethnicity) (%)	58.3%	64%	0.115
Years of schooling	12.5 ± 4.1	10.9 ± 4.2	<0.001
Household income (Brazilian reais)	6.018 ± 7.825	3.298,41 ± 3.257	<0.001
With partner (%)	80.6%	85.6%	0.082
Physical activity (%)	48.9%	36.4%	0.001
Smoking (%)	12.6%	11.9%	0.780
Alcohol consumption (%)*	50.4%	43.6%	0.065
Pregnancies**	2.6 ± 4.2	3 ± 1.7	0.188
Diabetes (%)	11.7%	21.6%	<0.001
Hypertension (%)	23.5%	42.8%	<0.001
Depression (%)	17%	24.6%	0.009

Vitória, Espírito Santo, Brazil, 2022 (*N* = 1.041).

*T*-test; chi-square; *N = 1.035 (404/102); **N = 821 (620/201).

Table 2 reveals that obesity prevalence is significantly higher among women aged 30–39 years, those with 0–8 years of schooling, those belonging to the poorest tertile of monthly household income, those who practiced physical activity, those who reported five or more pregnancies, and those who reported diabetes, hypertension, and depression (*p* < 0.05).

**Table 2:** Distribution of obesity according to sociodemographic, behavioral, and clinical factors in obese women living in the municipality of Vitória, Espírito Santo, Brazil, 2022 (*N* = 236).

Variables	Obesity (BMI ≥ 30 kg/m^2^)			
	*N*	*P*	95%CI	*p-*value
**Age group**				**0.021**
18–29 years old	29	15.3	10.8–21.2	
30–39 years old	54	27.6	21.7–34.2	
40–49 years old	55	25.7	20.3–34.3	
50–59 years old	48	25.4	19.7–32.1	
60 years or older	50	19.8	15.3–25.1	
**Ethnicity/skin color**				**0.115**
White	85	20.2	16.6–24.3	
Non-white	151	24.4	21.1–27.9	
**Years of schooling**				**<0.001**
0–8	65	33.9	27.5–40.9	
9–11	89	26	21.6–30.9	
12 or over	82	16.2	13.2–19.7	
**Household income**				**<0.001**
One tertile (poorest)	120	30.5	26.1–35.2	
Two tertile	78	23.4	19.1–28.2	
Three tertiles (richest)	38	12.1	8.9–16.3	
**Marital status**				**0.082**
Single	34	17.9	13.1–24.0	
In a relationship	202	23.7	21.0–26.7	
**Physical Activity**				**0.001**
No	86	17.9	14.7–21.6	
Yes	150	26.7	23.2–30.6	
**Currently smoking**				**0.780**
No	208	22.8	20.2–25.7	
Yes	28	21.7	15.4–29.7	
Current alcohol consumption*				**0.360**
Never	132	25,00	21.4–28.8	
Once a month or less	52	21.8	17.0–27.5	
2 to 4 times a month	30	17.9	12.8–24.4	
2 to 3 times a week	18	20.7	13.4–30.6	
≥ 4 times a week	2	16.7	3.9–49.5	
**Number of pregnancies**				**<0.001**
None	35	15.9	11.6–21.4	
1–2	88	18.6	15.4–22.4	
3–4	86	30.7	25.6–36.4	
5 or more	27	39.7	28.8–51.8	
**Diabetes**				**<0.001**
No	185	20.7	18.1–23.4	
Yes	51	35.2	27.8–43.3	
**Hypertension**				**<0.001**
No	135	18	15.4–20.9	
Yes	101	34.8	29.6–40.5	
**Depression**				**0.009**
No	178	21.4	18.4–23.9	
Yes	58	29.7	23.7–36.6	

Source: elaborated by the authors; PR, prevalence ratio; 95%CI: 95% confidence interval; *N = 234.

Previously, the variables were tested using a VIF in the raw analyses, with results below two being presented, indicating the absence of concerning collinearity. Table 3 presents a crude analysis of the prevalence ratio of obesity (BMI > 30 kg/m^2^) by sociodemographic, clinical, and behavioral characteristics. It was observed that the prevalence of obesity is higher in the 30–39 year (PR = 1.80; 95% CI: 1.20–2.69), 40–49 years (PR = 1.68; 95% CI: 1.12–2.51) and 50–59 year (PR = 1.66; 95% CI: 1.09–2.51) groups, with a decrease in the prevalence with age, compared to those aged 18–29 years. However, this trend is not observed in individuals aged 60 years or older (*p* = 0.044). Additionally, women with 0–8 and 9–11 years of schooling have 2.09 times (95% CI: 1.58–2.77) and 1.61 times (95% CI: 1.23–2.75) higher prevalences of obesity, respectively, compared to women with 12 or more years of schooling (*p* < 0.001).

**Table 3:** Crude analysis of the prevalence ratio of obesity according to the sociodemographic, clinical, and behavioral characteristics of women living in the municipality of Vitória, Espírito Santo, Brazil, 2022 (*N* = 1.041).

Variables	Obesity (BMI ≥ 30 kg/m^2^)		
	PR	95%CI	*p*-value
**Age group**			**0.027**
18–29 years old	**Ref.**		
30–39 years old	1.80	1.20–2.69	
40–49 years old	1.68	1.12–2.51	
50–59 years old	1.66	1.09–2.51	
60 years or older	1.29	0.85–1.96	
**Ethnicity/skin color**			**0.118**
White	**Ref.**		
Non-white	1.21	0.95–1.53	
**Years of schooling**			**<0.001**
0–8 years	2.09	1.58–2.77	
9–11 years	1.61	1.23–2.10	
12 or over	**Ref.**		
**Household income in tertile**			**<0.001**
First tertile (poorest)	2.51	1.80–3.50	
Second tertile	1.92	1.35–2.75	
Third tertile (richest)	**Ref.**		
**Marital status**			**0.091**
Single	**Ref.**		
In a relationship	1.33	0.96–1.84	
**Physical activity**			**0.001**
No	1.49	1.18–1.89	
Yes	**Ref.**		
**Smoking**			**0.781**
No	**Ref.**		
Yes	0.95	0.67–1.35	
Current alcohol consumption*			**0.379**
Never	**Ref.**		
Once a month or less	0.87	0.66–1.16	
2 to 4 times a month	0.72	0.50–1.02	
2 to 3 times a week	0.83	0.54–1.28	
≥ 4 times a week	0.67	0.19–2.39	
Pregnancies**			**<0.001**
None	**Ref.**		
1–2	1.17	0.82–1.68	
3–4	1.93	1.36–2.74	
5 or more	2.50	1.64–3.81	
**Diabetes**			**<0.001**
No	**Ref.**		
Yes	1.70	1.32–2.20	
**Hypertension**			**<0.001**
No	**Ref.**		
Yes	1.94	1.56–2.41	
**Depression**			**0.007**
No	**Ref.**		
Yes	1.41	1.10–1.82	

Source: elaborated by the authors; PR, prevalence ratio; 95%CI: 95% confidence interval; *N = 1.035; **N = 1.040.

Not engaging in physical activity increases the prevalence of obesity by 1.49 times (95%CI 1.18–1.89). Reproductive history is also a key factor; women with three to four pregnancies (PR = 1.93; 95% CI: 1.36–2.74) and five or more pregnancies (PR = 2.50; 95% CI: 1.64–3.81) demonstrate significantly elevated prevalence of obesity compared to those with no pregnancies. There seems to be a dose–response in which the probability of obesity grows as the number of pregnancies rises. Associations with obesity were also found in the presence of diabetes and hypertension when compared to women who did not present such factors (*p* < 0.001). Depression is another notable factor, with women experiencing this condition having a 59% higher frequency of obesity (PR = 1.41; 95% CI: 1.10–1.82). In contrast, behavioral factors such as smoking and alcohol consumption did not show significant associations with obesity in this analysis.

After adjustments (Table 4) for age group, ethnicity/skin color, years of schooling, household income, physical activity, pregnancies, diabetes, hypertension, and depression, it was observed that the associations were maintained. The effects of the mediation of the variables diabetes, hypertension, and depression were not significant, reinforcing the associations found. The probability of developing obesity was approximately 1.91 times higher in women aged 30–39 years, and it decreased with increasing age group (*p* = 0.006). Educational attainment and income levels exhibit strong inverse relationships with obesity prevalence (*p* < 0.05). Regarding pregnancy, only women who reported five or more pregnancies were more probability to show obesity compared to those who did not become pregnant (5 or more: PR: 1.66; 95%CI 1.04–2.64, *p* < 0.002). The frequency of diabetes, hypertension, and depression was also significantly associated with obesity compared to women without these diseases (*p* < 0.05). The association for physical activity was lost after adjustment.

**Table 4:** Adjusted analysis of the prevalence ratio of obesity with sociodemographic, behavioral, and clinical factors in women living in the city of Vitória, Espírito Santo, Brazil, 2022.

Variables	Obesity (BMI ≥ 30 kg/m^2^)		
	PR	95%CI	*p*-value
**Age group**			**0.006**
18–29 years old	**Ref.**		
30–39 years old	1.91	1.27–2.87	
40–49 years old	1.75	1.16–2.64	
50–59 years old	1.54	1.01–2.37	
60 years or older	1.21	0.78–1.87	
**Ethnicity/skin color**			**0.359**
White	**Ref.**		
Non-white	0.89	0.70–1.14	
**Years of schooling**			**0.009**
0–8 years	1.70	1.21–2.38	
9–11 years	1.31	0.99–1.75	
12 or over	**Ref.**		
**Monthly household income**			**0.002**
First tertile (poorest)	2.00	1.36–2.95	
Second tertile	1.73	1.20–2.51	
Third tertile (richest)	**Ref.**		
**Marital status**			**0.161**
Single	**Ref.**		
In a relationship	1.26	0.91–1.75	
**Physical activity**			**0.204**
No	1.17	0.92–1.50	
Yes	**Ref.**		
**Pregnancies**			**0.002**
None	**Ref.**		
1–2	0.94	0.65–1.36	
3–4	1.43	0.98–2.00	
5 or more	1.66	1.04–2.64	
**Diabetes**			**0.030**
No	**Ref.**		
Yes	1.34	1.03–1.74	
**Hypertension**			**0.001**
No	**Ref.**		
Yes	1.56	1.21–2.00	
**Depression**			**0.003**
No	**Ref.**		
Yes	1.44	1.13–1.83	

Source: elaborated by the authors; PR, prevalence ratio; 95%CI: 95% confidence interval.

Level 1 adjustment: age group, ethnicity/skin color, years of schooling, and household income.

Level 2 adjustment: age group, years of schooling, household income, and physical activity.

Level 3 adjustment: age group, years of schooling, household income, pregnancies, diabetes, hypertension, and depression.

## Discussion

The prevalence of overweight was found in more than half of the sample (55.2%); of these, almost half were obese (22%). A study conducted in six European countries, including two low- to middle-income countries (LMICs) and four socioeconomically vulnerable areas of high-income countries, reported total rates of overweight and obesity of 34.5 and 15.8%, respectively. Obesity rates were highest in Bulgaria and Hungary, which are LMICs, as well as in Greece, with Hungary having the highest rates (19.7%) (16). Obesity was 42.8% in the United States between 2017 and 2018 (17). In Brazil, according to the 2019 PNS (7), the prevalence of overweight among adult women was 62.6%, while obesity was observed in 29.5% of cases, being higher in this group compared to men, and obesity predominated in the 40–59 age groups (38%) (7). Comparing the findings with the self-reported data from Vigitel (2019) for the national population (18), the frequency is 53.9% for overweight and 20.3% for obesity, with overweight being more frequent among men and obesity equally distributed between sexes but higher in the 45–54 age group of women (25.2%; 95% CI 23–27.4). In Vitória, the prevalence of overweight and obesity among women was 47.8% (95%CI 43.8–51.7) and 19.1% (95%CI 16.2–22.0), respectively. Data from the same study in 2013 showed a prevalence of 16.1% (95%CI 13.3–18.8) in obesity (8), demonstrating an increasing trend over time.

It is essential to highlight the presence of information bias, with underestimation and overestimation of weight and height in self-reported measurements. Younger women (<40 years) and those with higher education levels tend to underestimate their weight, while older women with lower education levels tend to overestimate it (19). However, most studies indicate an underestimation of the BMI in women when using self-reported data (20, 21), being more common among women with obesity compared to those with normal weight (22). A review of different studies indicates that, in most cases, nutritional status is not affected by minor variations despite current estimates of obesity prevalence based on self-reported data being underestimated (21). Nevertheless, self-reported weight and height are sufficient for assessing nutritional status, allowing their use in association studies and as outcome variables (19, 22, 23).

The difference in prevalence can be explained by the applied methodologies and territorial coverage since the PNS uses assessed anthropometric information and refers to national data (7). However, despite the issues mentioned, the data are similar when it is observed that more than half of the female population is overweight, and, of these, almost half are obese. Bhaskaran et al. (14) observed an association between BMI ≥ 25 kg/m2 and increased all-cause mortality. In addition, the loss of years of quality of life is highlighted, with obesity propelling most of the burden of absolute mortality. Obesity reduces healthy life years, increases mortality, and represents a growing economic burden on health (14). The data are concerning, as nearly one-quarter of the female population in the city of Vitória is affected by obesity. These findings are consistent with those of other studies, highlighting obesity as not only a public health issue but also a social and economic challenge.

The data from our study show an inverse association between schooling and family income; as they increased, the prevalence of obesity decreased. The research conducted in Europe corroborates this finding, with a higher obesity prevalence (18.9%) observed among individuals with lower education levels (< 12 years of schooling) compared to those with more than 12 years of education (14.2%) (16). In Latin American countries, a higher prevalence of obesity is found among women with lower educational levels compared to those with higher levels. This differed from men, where education was not associated, raising important gender issues (24). A study using data from Argentina’s 4th National Survey of Risk Factors among people with low socioeconomic status shows that obesity disparities in the country were pronounced for women but not for men (25). The findings corroborate those of Ferreira et al. (26), who found that the most significant increases in obesity occurred in women with fewer years of schooling and lower household income, despite the rise in obesity in all groups. There was also a higher frequency of obesity in women (26.5%; 95% CI 24.7–28.3) who had 0 to 8 years of schooling and lived in Vitória, ES (20). It was observed that Brazilian women with up to 7 years of education had a higher prevalence of obesity (29%; 95% CI 27.3–30.8) compared to those with 15 or more years of schooling (18.5%; 95% CI 16.1–21.0) (27).

Regarding household income, a study conducted in the U.S. evaluated economic conditions and observed a significant increase in the average BMI across all classes. However, the overall BMI was higher among individuals with lower economic conditions than those with better economic conditions. It is noteworthy, however, that the increase in obesity prevalence was primarily attributed to the rise among those with better economic conditions. This raises a reflection on access to food and helps to explain the higher obesity rates in higher-income countries, as well as the growing prevalence of obesity in LMICs as these countries develop. The health costs associated with this disease, in addition to the impacts on mortality and loss of quality of life, compromise the overall development of LMICs (17).

Other studies have shown a positive relation between parity and increased BMI (28, 29), which is consistent with our findings. Women with five or more pregnancies were more probability to show obesity compared to those who did not become pregnant. This indicates an association between the number of pregnancies and nutritional status; the higher the number, the greater the probability.

In this study, the prevalence of diabetes mellitus was 13.8% among women in general (95% CI 11.9–16.0) and 34% (95% CI 27.2–42.5) among obese women. Compared to Vigitel for the same city, the prevalence of diabetes mellitus in the population in 2013 was 7.8% (95% CI 6.2–9.4) and 13.1% (95% CI 7.6–18.6) in PNS (8). A population-based study with women conducted in 2015 in the South region of the country, with self-reported data, described a prevalence of 8.16% (95% CI 2.56–13.74) of diabetes mellitus, with the prevalence being 41% higher among obese women than among eutrophic women (30). In Brazil, in 2019, the state of Espírito Santo stood out with one of the most significant increases in the prevalence of diabetes mellitus. However, these numbers should be carefully evaluated since they may have occurred due to better access to health services, considering that the rise was concentrated in the states of the Southeast region (31).

The prevalence of women with hypertension in the sample was 27.3% (95% CI 24.7–30.1) and almost 30% higher in obese women than in women in general (34.5%; 95% CI 29.2–40.1). Compared to Vigitel, the frequency of hypertension in 2013 was 27.9% (95% CI 25.1–30.7), while in the PNS, it was 25.2% (95% CI 20.0–30.05) (8). More recent data from Vigitel for the municipality indicate 28.1% (95% CI 25.0–31.1) (19). The systematic review pointed to obesity as the main anthropometric factor associated with arterial hypertension, corroborating our finding of a higher frequency of obesity in women who have this disease compared to those without (32).

Although this is a cross-sectional study, it is essential to emphasize that obesity is one of the main contributors to the development of hypertension worldwide, and not the other way around, even though obesity-induced increases in blood pressure are multifactorial (33). Studies have shown that obesity is causally related to hypertension and diabetes mellitus, especially type 2, among other cardiovascular and kidney diseases (34, 35).The impact of obesity, in addition to other chronic diseases such as hypertension and diabetes mellitus, results in an increase in health services, a greater demand for human resources and materials, and, consequently, a more significant burden on the national health system (36).

Depression and obesity have also been widely explored. A meta-analysis study demonstrates the bidirectional relationship between depression and obesity, showing a positive association between the two. Among the eight studies analyzed, obesity was associated with a 1.57 times higher likelihood of depression, even after adjustments (37). This finding aligns with our results, showing a higher frequency of obesity in women with depression compared to those with a normal BMI (38).

In this context, sociodemographic factors such as age, family income, and education level have been consistently identified as determinants of obesity. Behavioral factors, including physical inactivity, compound the problem, particularly in women. The presence of chronic diseases such as diabetes, hypertension, and depression has also been shown to exacerbate the negative health consequences of obesity. It should be emphasized that the association with physical activity was not found in our study, which may be considered a limitation, as the question used to measure physical activity did not account for the type, duration, or frequency of physical activity.

Among the positive aspects of the present study is that it is a population-based study that is representative of women living in the city of Vitória. These findings are highly relevant for comparison with existing data, allowing for assessing trends and obesity behavior over time in this specific urban context. Our results corroborate existing evidence in the literature, providing additional robustness and precision and contributing to consolidating the scientific knowledge base on this subject.

A potential limitation of the study is the use of self-reported anthropometric data, which may be subject to information bias, even with data collection through face-to-face interviews, as discussed earlier. The cross-sectional design is also a limiting component since the exposure to risk factors, such as income, education, marital status, diabetes, hypertension, and depression, may have been modified after the development of obesity, which makes it challenging to establish exposure as effectively preceding the outcome. Despite the large number of studies on the topic, obesity continues to show an upward trend, and current strategies to address it have proven insufficient, reinforcing the timeliness and urgency of further investigation.

The data corroborate findings of similar methodology and guide the female population’s health situation. The findings indicate that obesity is a growing and important public health problem in the female population and should be urgently addressed. Women’s greater vulnerability to obesity is highlighted, driven by social determinants of health that affect men and women in different ways. In conclusion, obesity is associated with age, household income, years of schooling, number of pregnancies, and chronic diseases such as diabetes, hypertension, and depression. It is essential to understand the determinants of obesity in women and gender differences as a necessary part of public health policies and new strategic actions for prevention and treatment, aiming to reduce disparities in non-communicable diseases. Identifying the most vulnerable groups in an urban Brazilian setting can inform more targeted local and regional interventions, supporting the planning of intersectoral actions within the Brazilian Unified Health System (SUS), as well as the development of specific public policies for health promotion, given the complexity of the obesity phenomenon.

In this sense, to effectively combat obesity among women, structural changes in the built environment must be prioritized to promote physical activity, including developing safe and accessible public spaces. Additionally, educational interventions should be designed and implemented to enhance health literacy, particularly among socioeconomically disadvantaged groups. Furthermore, other critical social measures must not be overlooked to support a healthy environment for women with children. Multi-sectoral collaborations involving healthcare providers, policymakers, and community leaders must be strengthened to ensure the effectiveness and sustainability of these interventions.

## Data Availability

The original contributions presented in the study are included in the article/supplementary material, further inquiries can be directed to the corresponding author.
